# Automated recognition and analysis of head thrashes behavior in *C. elegans*

**DOI:** 10.1186/s12859-022-04622-0

**Published:** 2022-03-07

**Authors:** Hui Zhang, Shan Gao, Weiyang Chen

**Affiliations:** 1 School of Computer Science and Technology, Qilu University of Technology (Shandong Academy of Sciences), Jinan, 250353 China; 2 Beijing Center for Disease Prevention and Control, Beijing Key Laboratory of Diagnostic and Traceability Technologies for Food Poisoning, Beijing, 100013 China

**Keywords:** *Caenorhabditis elegans*, Head thrashes, Image processing, Automated count

## Abstract

**Background:**

Locomotive behaviors are a rapid evaluation indicator reflecting whether the nervous system of worms is damaged, and has been proved to be sensitive to chemical toxicity. In many toxicological studies, *C. elegans* head thrashes is a key indicator of locomotive behaviors to measure the vitality of worms. In previous studies, the number of head thrashes was manually counted, which is time-consuming and labor-intensive.

**Results:**

This paper presents an automatic recognition and counting method for head thrashes behavior of worms from experimental videos. First, the image processing algorithm is designed for worm morphology features calculation, mean gray values of head and tail are used to locate the head of worm accurately. Next, the worm skeleton is extracted and divided into equal parts. The angle formulas are used to calculate the bending angle of the head of worm. Finally, the number of head thrashes is counted according to the bending angle of the head in each frame. The robustness of the proposed algorithm is evaluated by comparing the counting results of the manual counting. It is proved that the proposed algorithm can recognize the occurrence of head thrashes of *C. elegans* of different strains. In addition, the difference of the head thrashes behavior of different worm strains is analyzed, it is proved that the relationship between worm head thrashes behavior and lifespan.

**Conclusions:**

A new method is proposed to automatically count the number of head thrashes of worms. This algorithm makes it possible to count the number of head thrashes from the worm videos collected by the automatic tracking system. The proposed algorithm will play an important role in toxicological research and worm vitality research. The code is freely available at https://github.com/hthana/HTC.

## Background

Caenorhabditis elegans (*C. elegans*) is a small, common worm that can live freely in the soil. The adult worms are about 1 mm long and feed on bacteria. Compared with other model organisms, it is characterized by small size, strong reproductive capacity, short and accurate life cycle, multiple sensitive detection indicators [1], easy cultivation in laboratory, conservative genetic background, low cost, easy preservation, fast passage and transparent body. Because of these properties, *C. elegans* is widely used in many areas of biology, including aging, behavior, neuroscience and development [2–5]. *C. elegans* is one of the most widely used model organisms in the field of life science. Because of its sensitivity to exogenous compounds, *C. elegans* has unique advantages in multi-generation toxicity studies. *C. elegans* have been used in toxicity assessment and toxicological studies of many toxicants, including organic pollutants [6–8], metals [9, 10], pesticides [11–13] and medical drugs [14–16]. For example, Li et al. studied the plasticizer di(2-ethylhexyl) phthalate (DEHP), an emerging organic pollutant in environmental science. By studying the locomotive behaviors of worms exposed to DEHP for a long period, it is demonstrated that long-term exposure to DEHP leads to multigenerational defects in locomotive behaviors, increasing potential health and ecological risks [6]. Wang et al. studied the combined behavioral toxicity of multiple pesticides and their binary mixtures to worms. The toxicity of various pesticides was assessed by locomotive behaviors such as head thrashes and body bends. The results showed that the four insecticides and their binary mixed rays could significantly inhibit the locomotive behavior of worms [11].

Locomotive behaviors are a rapid evaluation indicator reflecting whether the nervous system of worms is damaged [17], and has been proved to be sensitive to chemical toxicity [1]. In many toxicological studies, worm head thrashes frequency and body bends frequency were selected as two locomotive behaviors indicators to measure the lifespan and vitality of worms [6–16]. In the research of locomotive behaviors of worms, thrashing frequency was defined as the number of wavelengths that the worm moved through in 1 min [18]. In previous studies, the number of head thrashes was manually counted, which is time-consuming and labor-intensive. In addition, when the worm’s head thrashes fast, the manual counting would result in some error.

In this paper, an automation counting method for counting head thrashes of worms is presented. After selecting worm video randomly from *C. elegans* behavioral phenotype database [19], the video is first divided into frames and each frame of gray image is preprocessed to segment worm body. The proposed algorithm combines binary image and gray image to increase the accuracy of head recognition. According to three criteria “the worm’s head is rounder than the tail”, “the worm’s tail is darker than the head” and “the head distance between two consecutive frames”, image processing algorithm for calculation of worm morphology features and calculation of mean gray values of head and tail are used to locate the head of worm accurately. Next, the worm skeleton is extracted and marker points are placed to divide worm skeleton equally. The angle formulas are used to calculate the bending angle of the head. Finally, the number of head thrashes is counted according to the bending angle of the head in each frame. In addition, we test parameters related to the number of head thrashes of worms with different lifespans and analyze worm vitality. At the same time, the proposed algorithm can be applied to toxicological research to reduce time and labor consumption.

## Methods

**Fig. 1:**
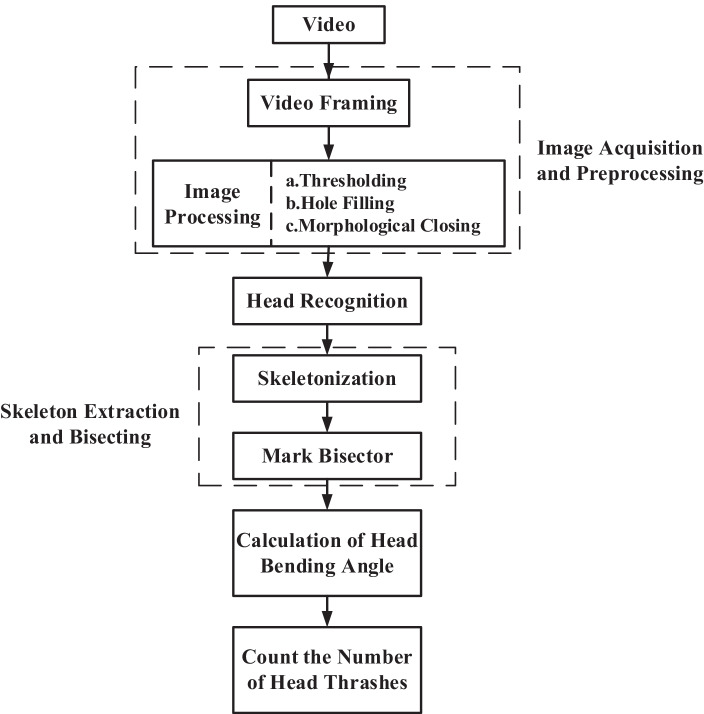
The general description of the proposed algorithm implementation process

In this section, the proposed algorithm is described in detail. Firstly, the video is segmented into frames to get the original gray image, the gray image is preprocessed to detect the worm. Secondly, the binary image and gray image are combined to recognize the worm’s head. Thirdly, the worm skeleton is extracted and divided into equal parts. Fourthly, the bending angle of head in each frame is calculated. Finally, the number of head thrashes of worms is counted from the head bending angle curve. The proposed algorithm flow chart is shown in Fig. 1.

### Image acquisition and preprocessing

The videos in the experiment are downloaded from *C. elegans* behavioral phenotype database [19]. In order to get the image data, the original gray images are extracted from video. The process of image preprocessing is shown in Fig. 1. Since the worms in the video behave spontaneously on food, the worms in the video have more shadows around them. In order to obtain a better binary image, the original grayscale image is selected for cutting, and then the maximum gray value of the image's four corners (at least one corner point always does not belong to the worm body) is obtained to determine the background intensity level of the original gray image. After the background level ($b$) of the image is determined, an adaptive local threshold algorithm is applied to the image. Firstly, a 5 × 5 moving window is used to scan the image and calculate the mean ($m$) and standard deviation ($s$) of pixels inside the window at each pixel position. If $m < 0.7b$ or $s > 0.3m$, the center pixel in the moving window is assigned to 1 as a part of the worm's body. Otherwise, the center pixel in the moving window is assigned a value of 0 as the background [20–22]. Next, a morphological closing operator (binary dilation followed by erosion) [23] is used to remove small spots in worms. Finally, the sequential algorithm for component labeling is used to remove unwanted isolated small objects [24]. Each pixel of the image is scanned in the *x* and *y* directions. Those with the same pixel value are divided into the same group, and the connected components are marked. Subsequently, the connected components of all pixels in the image are obtained. In order to ensure that there is only one object in the binary image, it is necessary to select the largest component among all components, the worm. In addition, in order to make subsequent processing more convenient, the binary image is reversed processing.

### Algorithm for head recognition

To increase the accuracy of recognizing the head, the proposed algorithm use three criteria. The first criterion is that the worm’s head is rounder than the tail; The second criterion is that the worm’s tail is darker than the head; The third criterion is based on the head distance between two consecutive frames [22]. Criterion 1 and 2 are used to recognize the worm’s head in the first frame of the video. Criterion 3 is used to recognize the worm’s head during the tracking of worm movement.

The worm’s head is rounder than the tail. First, the edge detection algorithm is used to detect the worm contour. Next, the detected worm contour data are smoothed to obtain the coordinate data of each edge point. Then, the contour points of the worm are re-sampled, that is, the fixed distance with a linear interpolation method is used to evenly sample the worm [25]. The contour is divided into equal distances, and the coordinate information of each partition point is recorded. The re-sampled points can be defined as $P_{i}$, $i$ = 1,…$n$, the sharpness of a boundary point relative to its neighbors can be calculated as

1 $$S_{k,i} = (P_{i + k} - P_{i} ) \cdot (P_{i - k} - P_{i} ) \doteq l_{k}^{2} \cos \theta_{i}$$

where $k$ is the index increment, $\theta_{i}$ is the acute angle between two intersection vectors, $l_{k}$ is the length of the vector corresponding to the index increment. A larger $S_{k,i}$ indicates a smaller $\theta_{i}$ and sharper boundary point. Since $S_{k,i}$ is the sharpest point on the worm body contour, the tail of the worm can be identified by calculating $S_{k,i}$. In order to avoid the deviation caused by $k$ value, the length of the size vector was combined and the worm tail point $P_{t}$ is defined as

2 $$t = \arg \mathop {\max }\limits_{i} \left\{ {S_{{l_{1,i} }} + \frac{{l_{1}^{2} }}{{l_{2}^{2} }} \cdot S_{{l_{2,i} }} } \right\},i \in \left\{ {1,...n} \right\}$$

The worm head point $P_{h}$ is defined as

3 $$h = \arg \mathop {\max }\limits_{i} \left\{ {S_{{l_{1,i} }} + l_{1}^{2} /l_{2}^{2} \cdot S_{{l_{2,i} }} } \right\},i \in \left\{ {1,...n} \right\} - \left\{ {t - w,...t + w} \right\}$$

where $w$ is the region width to exclude the tail area. Here, we set $w = n/4$, $l_{1} = n/40$, and $l_{2} = n/100$.

The worm’s tail is darker than the head. In order to increase the accuracy of head recognition, the proposed algorithm recognizes the worm’s head by gray value according to the coordinates of head and tail calculated in the first criterion. First, the coordinate index of the head and tail calculated in binary image is used as the coordinate index of the original gray image. Next, the proposed algorithm divided the two points and the surrounding points into two groups and calculated the median brightness of the two endpoints. Compare the mean values of the two groups, and if the difference between the two means is at least 10% of the larger mean, then the group with the higher mean brightness value and the corresponding endpoint is the worm’s head.

**Fig. 2:**
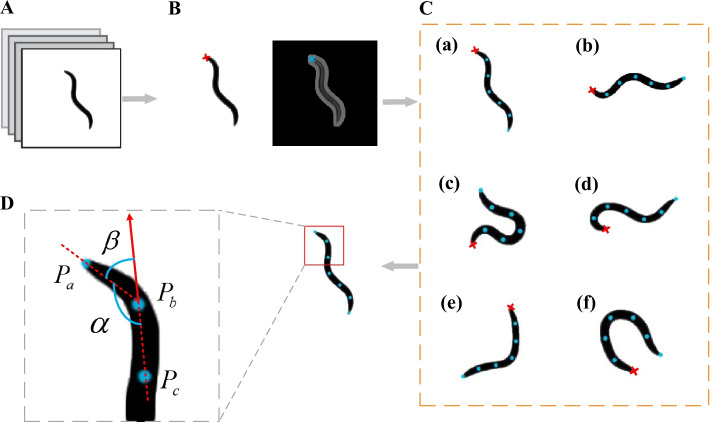
**A**–**D** Calculation process of head bending angle. **A** Binary images after preprocessing. **B** Result graph of head recognition. The figure on the left recognizes the head by calculating the worm morphological features; The figure on the right recognizes the head by calculating the average gray value of the head and tail of the worm. **C** Marker points were placed to divide the worm skeleton equally. (a–f) Different phenotypes of worms were selected to place marker points and divide the skeleton equally. **D** Calculation of bending angle of head

The result of head recognition is shown in Fig. 2B. The recognition results of the two methods were compared. If the recognition results are consistent, the proposed algorithm proceeds to the next step. If the recognition results are inconsistent, the resulting graph recognized by the two methods will be displayed, and the user will be prompted whether to exchange the head and tail coordinates. According to the user's judgment, if the result is consistent with the first criterion, there is no need to exchange the head and tail coordinates; If the result is consistent with the second rule, change the head and tail coordinates and proceed to the next step.

In each subsequent frame, the head and tail coordinates of the previous frame are used as the reference standard. For example, in recognizing the head of the second frame, the distance between the head coordinates of the first frame and the two endpoints obtained after the skeleton extraction of the second frame is calculated separately. As mentioned in Ref. [22], among the four offsets of head and tail in worm coordinates for two consecutive frames, the corresponding offsets of head-head and tail–tail are the smallest. Therefore, the endpoint with the shortest distance between the head of frame 1 and the endpoint of frame 2 is the worm’s head.

### Skeleton extraction and bisecting

To simplify the calculation, the skeleton of the worm is extracted. First of all, we have obtained the coordinates of worm contour points in the previous step and calculated the midpoint coordinates through the two points on the dorsal and ventral sides of the worm. Next, the cubic spline interpolation of the midpoint is performed to obtain the spline curve, which is the skeleton of the worm [26]. Then, the worm is divided into $N$ segments by placing $N + 1$ marker points isometric (the value of $N$ is determined by the degree of bending of the head of the worm). In this paper, different phenotypes of worms are selected to place marker points and divide the skeleton equally. The resulting graph is shown in Fig. 2C. Six representative phenotypes are shown and according to these marks, the bending angle of head can be calculated accurately. Here, we set $N$ = 7.

### Calculation of head bending angle

The worm is divided equally by placing marker points, and the first three points from the head are selected to calculate the bending angle of the head. A schematic diagram for calculating the bending angle of the worm head is shown in Fig. 2D.

Define three points as $P_{a}$,$P_{b}$ and $P_{c}$. According to the law of cosines $\alpha$ can be calculated as

4 $$\alpha = \arccos \left( {\left( {l_{a}^{2} + l_{b}^{2} - l_{c}^{2} } \right)/2 \cdot l_{a} \cdot l_{b} } \right)$$

where $l_{a}$ is the distance between $P_{a}$ and $P_{b}$, $l_{b}$ is the distance between $P_{b}$ and $P_{c}$, $l_{c}$ is the distance between $P_{a}$ and $P_{c}$. The worm head thrash angle $\beta$ can be calculated as

5 $$\beta = \pi - \alpha$$

In the case of excessive head bending in Fig. 2C(d), the angle calculated by these formulas is more than 90. In order to avoid deviation in counting the number of head thrashes, the angle value more than 90 was set to 90.

### Count the number of head thrashes

**Fig. 3:**
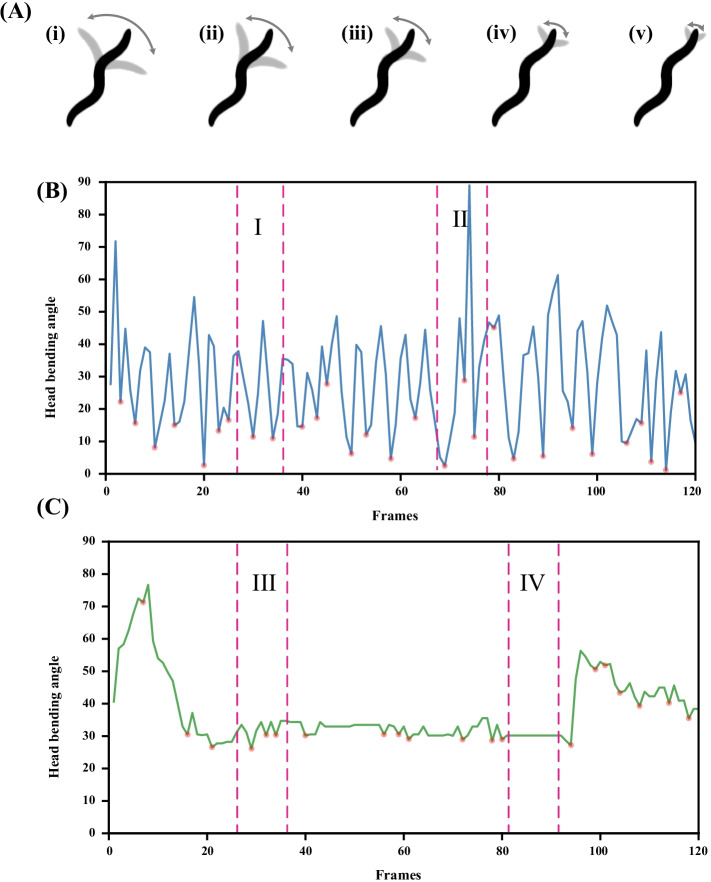
**A** The graph of the head thrashes of worm. From (i) to (v), the amplitude of head thrashes decreased gradually. The situation shown in (v) may be a foraging behavior. The proposed algorithm reduce the bias caused by foraging behavior by setting a threshold. (**B**, **C**) Head bending angle curves. The red point is the inflection point where the bending angle of head changes from large to small and then becomes large again, and it is also the reference point for calculating the number of head thrashes. In area II of **B**, head thrashes of worm violently, with the head bending angle ranging from 0 to 90 and the maximum head bending angle reaching 90. In area I of **B**, head thrashes of worm frequently, with the head bending angle ranging from 10 to 50. In area III of **C**, head thrashes of worm slightly, with the head bending angle ranging from 25 to 35. In area IV of **C**, straight line appears in the figure, this indicated that the head of worm remained stationary during this period

In previous studies, head thrashes were defined as one head thrashing when the body bending of worm reached half of its body length [18]. This counting method has some limitations, it cannot be calculated for the small amplitude of the head thrashes. In Fig. 3A(i), the worm’s head is bent up to half its body length; In Fig. 3A(ii), the worm’s head is bent up to one-third of its body length; In Fig. 3A(iii), the head of the worm is bent up to a quarter of its body length; In Fig. 3A(iv), the head of the worm is bent up to one-sixth of its body length. For the various head thrashes cases shown in Fig. 3A, there will be a large deviation in the results calculated by only referring to the judgment criteria in Ref. [18].

Herein, the worm’s head from side to side and back again is recorded as a head thrash. First, the head and tail positions of worms in the first frame are recognized, worm skeleton is extracted, marker points were set to divide worm skeleton equally, and the bending angle of head is calculated by using the first three points from the head. Then, in each subsequent frame, the head and tail coordinates of the previous frame are used as the reference standard. For example, in recognizing the head of the second frame, the distance between the head coordinates of the first frame and the two endpoints obtained after the skeleton extraction of the second frame is calculated separately. As mentioned in Ref. [22], among the four offsets of head and tail in worm coordinates for two consecutive frames, the corresponding offsets of head-head and tail–tail are the smallest. Therefore, the endpoint with the shortest distance between the head of frame 1 and the endpoint of frame 2 is the worm’s head.

Each subsequent frame needs to calculate the bending angle of head according to the first frame standard. Finally, the number of head thrashes of worm is calculated according to the change of angle. The curve of angle change is shown in Fig. 3. The red point is the inflection point where the bending angle of head changes from large to small and then becomes large again, and it is also the reference point for calculating the number of head thrashes. In area II of Fig. 3B, head thrashes of worm violently, with the head bending angle ranging from 0 to 90 and the maximum head bending angle reaching 90. In area I of Fig. 3B, head thrashes of worm frequently, with the head bending angle ranging from 10 to 50. In area III of Fig. 3C, head thrashes of worm slightly, with the head bending angle ranging from 25 to 35. In area IV of Fig. 3C, straight line appears in the figure, this indicated that the head of worm remains stationary during this period.

In addition, worms rapidly wiggle their noses to explore the environment during foraging [27]. The proposed algorithm reduces the bias caused by foraging behavior by setting a threshold value, calculating the angle difference between two consecutive frames, putting it into an array, iterating over the number array and removing the value with an absolute value less than 5.

## Results

In this section, experimental verification is performed to verify the effectiveness of the proposed algorithm. First of all, experimental verification is performed to verify the accuracy of the head recognition algorithm. Secondly, the robustness of the proposed algorithm is evaluated by comparing the counting results of the manual counting. To be specific, a trained human observer is selected to count the number of head thrashes of the worm. Then, to exploit the relationship between the vitality of worms with lifespan, the number of head thrashes of different worm strains is counted by the proposed algorithm and the results are analyzed and discussed.

### *C. elegans* strains

Wild-type (Schafer Lab N2, Bristol) and 6 mutants *ser-1(ok345)*, *daf-7(m62)*, *egl-8(n488)*, *daf-5(e1386)*, *ser-4(ok512)* and *unc-10(md1117)* of *C. elegans* are obtained from *C. elegans* behavioral phenotypes database [19]. Worms culture methods and video data acquisition are described as [19]. In these experiments, all the worms used in the analysis are young adults, spontaneously behaving on food. Before video data of worms were collected, all worms were kept under strictly controlled conditions [2]. And the worms were picked and moved to their tracking plate to acclimate for 30 min before being tracked. In the process of collecting worms video data, in order to improve the resolution of the video taken, the camera magnification was set between 3.5–4.5 microns/pixel (a corresponding FOV of, approximately, 2.5 × 2 mm at 640 × 480 resolution). At the same time, to avoid potential indoor conditions leading to measurement bias, the recording was distributed as randomly as possible across multiple trackers. The frame rate of the video in the *C. elegans* behavioral phenotype database [19] is 20–30 frames per second.

### Head recognition

The head and tail recognition algorithm is tested on the first frame of 210 1-minute videos from 7 mutant types. During the experiment, the algorithm marks the worm's head for verification by human observers. The experimental results are shown in Table 1. The rate of conflict between curvature-based and grayscale-based head recognition methods is 3.8%. If the grayscale-based method is used as the judgment criterion when conflicts occur, the final head recognition error rate is around 2.9%. Manual checking is used to ensure that the head recognition in the first frame is correct when conflicts occur. Because in each subsequent frame, the head and tail coordinates of the previous frame are used as the reference standard. For example, in the head recognition of the second frame, the distance between the head coordinates of the first frame and the two endpoints obtained after the skeleton extraction of the second frame is calculated respectively. As mentioned in Ref. [22], among the four offsets of head and tail in worm coordinates for two consecutive frames, the corresponding offsets of head-head and tail–tail are the smallest. Therefore, the endpoint with the shortest distance between the head of frame 1 and the endpoint of frame 2 is the head of worm. By using this method, the head recognition accuracy of each subsequent frame could be guaranteed.

**Table 1:** Recognition of the head for various strains

Worm type	Number of videos	Number of conflicts^a^	Curvature-based wrong^b^	Grayscale-based wrong^c^	Recognition wrong^d^
*ser-1 (ok345)*	30	0	0	0	1
*daf-7 (m62)*	30	2	1	1	1
*egl-8 (n488)*	30	2	2	0	0
N2	30	0	0	0	0
*daf-5 (e1386)*	30	3	2	1	2
*ser-4 (ok512)*	30	0	0	0	0
*unc-10 (md1117)*	30	1	1	0	0
Total	210	8	6	2	4

^a^The number of conflict the between curvature-based and grayscale-based head recognition methods

^b^The error that occurred in curvature-based method when conflict occurs

^c^The error that occurred in grayscale-based method when conflict occurs

^d^The error that occurred in recognition of the head when no conflicts occur

### Algorithm verification by human observers

**Fig. 4:**
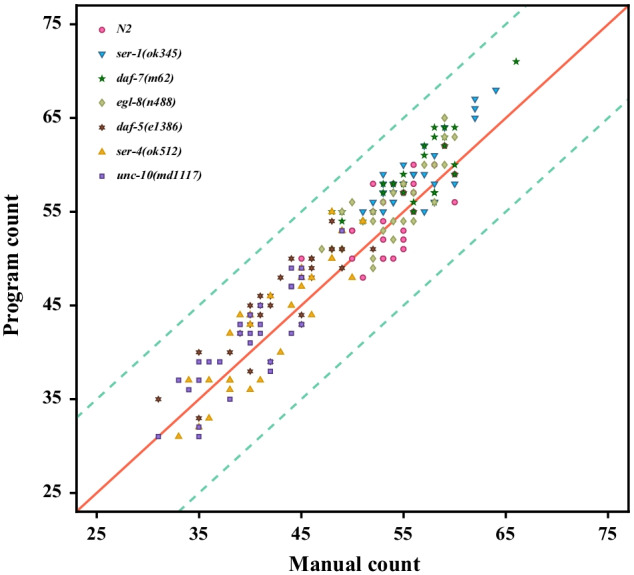
Results for counting the number of head thrashes manually and programmatically. The horizontal axis is the number of head thrashes is manually counted and vertical axis is the number of head thrashes is programmatically counted. The solid red line is a straight line with slope k = 1. The two green dotted lines are error lines with slope k = 1 and intercept b = 10. Dots of different colors and shapes represent different strains of worms

To evaluate the accuracy and robustness of the algorithm, the algorithm for automatically counting the number of head thrashes was tested in 210 1- minute videos. Wild-type (Schafer Lab N2, Bristol) and 6 mutants *daf-5(e1386)*, *daf-7(m62)*, *egl-8(n488)*, *ser-1(ok345)*, *ser-4(ok512)* and *unc-10(md1117)* of *C. elegans* are obtained from *C. elegans* behavioral phenotypes database [19]. Each strain has 30 videos. First, a trained human observer is selected to count the number of head thrashes in each video and record the results. Then the proposed algorithm is used to count the number of head thrashes in each video and record the results. Experimental results of manual count and program count are shown in Fig. 4. The results of manual count and program count show the linear distribution and the average absolute error is **3.0714**, the Pearson Correlation Coefficient between manual counting results and program counting results is **0.9463**, indicating that our algorithm is very robust.

### The difference of head thrashes frequency of different worm strains

**Fig. 5:**
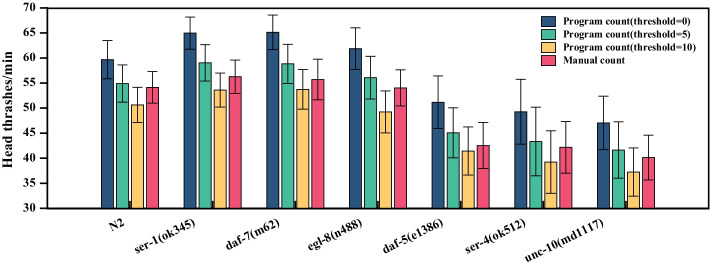
The mean and standard deviation of head thrashes frequency by manual and program count. The blue bar represents the program count results with threshold values is 0. The green bar represents the program count results with threshold values is 5. The orange bar represents the program count results with threshold values is 10. The red bar represents the manual count results

In order to analyze the difference of head thrashes frequency of different worm strains which have different lifespans, select N2 of *C. elegans*, the strains with longer lifespan are *ser-1(ok345)*, *daf-7(m62)* and *egl-8(n488)* of *C. elegans*, the strains with shorter lifespan are *daf-5(e1386)*, *ser-4(ok512)* and *unc-10(md1117)* of *C. elegans* [1, 17, 28–32]. We randomly select 30 groups of 1-min videos for worms of each strain and counted the number of head thrashes manually and programmatically. Then calculate the mean and standard deviation of manual count and program count of worms of each strain and the results are shown in Fig. 5. In this paper, the number of head thrashes were counted by setting a threshold value on the bending angles differences between consecutive frames. Figure 5 shows the program count results with various threshold values (0, 5 and 10). In the process of human eye observation, when the worm head thrashes frequently or with a small amplitude, manual counting is prone to error and human bias, resulting in a lower result of manual counting than program counting. In addition, the manual counting would lead to counting underestimation. To reduce this bias, the threshold value is set as 5 in the program count. The number of head thrashes of N2 of *C. elegans* is about 55 times per minute; The number of head thrashes of *ser-1(ok345)* of *C. elegans* is about 58 times per minute; The number of head thrashes of *daf-7(m62)* of *C. elegans* is about 58 times per minute; The number of head thrashes of *egl-8(n488)* of *C. elegans* is about 57 times per minute; The number of head thrashes of *daf-5(e1386)* of *C. elegans* is about 45 times per minute; The number of head thrashes of *ser-4(ok512)* of *C. elegans* is about 43 times per minute; The number of head thrashes of *unc-10(md1117)* of *C. elegans* is about 42 times per minute. The results showed that the head thrashes frequency of the long lifespan worm strains are higher than that of N2 of *C. elegans*, while the head thrashes frequency of the short lifespan worm strains are lower than that of N2 of *C. elegans*. Head thrashes behavior is a key indicator of locomotive behaviors in toxicological studies [6–8]. Vitality refers to the rate at which age-related physiological changes occur during an organism’s lifespan. Vitality is related to locomotory rates in worms. *C. elegans* shows age-related decline in vitality, which is manifested by reduced body locomotory [33–39]. The seven strains have the same culture conditions, and the experiment results show that the head thrashes frequency of the long-lived worms is higher than that of the short-lived worms. It can be inferred that the long lifespan worm strains show higher vitality.

**Fig. 6:**
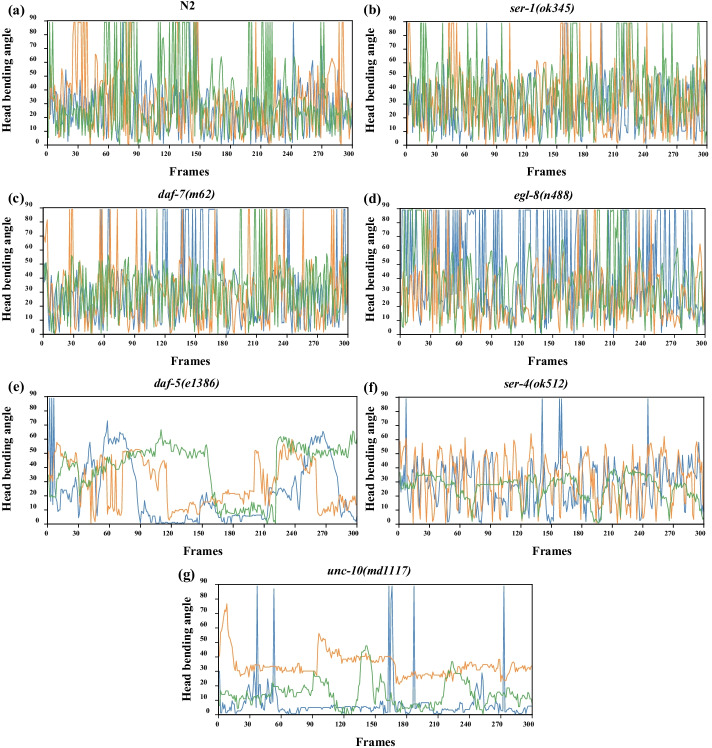
Head bending angles curves of different worm strains. In panel (**a**–**g**), the blue, green and orange curves are three groups of worms randomly selected from the same strain to show the head bending angles of worms

In order to better understand the difference in head thrashes frequency and amplitude between different strains of worms, we plot the head bending angles curve as shown in Fig. 6. Three 300 frames of video sequence are randomly selected from worms of each strain to plot the head bending angles curve. As can be seen from Fig. 6(a–d), the head bending angles of N2, *ser-1(ok345)*, *daf-7(m62)* and *egl-8(n488)* of *C. elegans* converge at 20 to 50 degrees. With a lot of great head bending close to 90 degrees. The head bending angle changes frequently between adjacent frames. It illustrates that head thrashes of N2, *ser-1(ok345)*, *daf-7(m62)* and *egl-8(n488)* of *C. elegans* are strong. These four strains show high vitality. As can be seen from Fig. 6(e–g), the head bending angles of *daf-5(e1386), ser-4(ok512) and unc-10(md1117) of C. elegans* show high variability. For example, as can be seen from Fig. 6(g), the blue curve is almost always horizontal and has a small bending angle from frames 90 to 150, indicating that the worm’s head is almost stationary during this period. The orange curve has a smaller oscillation amplitude, indicating that the head thrashes amplitude slightly and may be accompanied by foraging behavior during this period. But compared to the first four strains, the head bending angle changes slowly between adjacent frames. It illustrates that head thrashes *daf-5(e1386)*, *ser-4(ok512)* and *unc-10(md1117)* of *C. elegans* are variable, sometimes head thrashes of worm frequently, sometimes head thrashes of worm slightly, and sometimes the head of worm remained stationary. From a macro perspective, the head thrashes frequency of the last three strains was lower than that of the first four strains. The latter three strains show relatively low vitality.

## Discussion

A new method is proposed to automatically count the number of head thrashes of worms. This algorithm makes it possible to count the number of head thrashes from the worm videos collected by the automatic tracking system. A trained human observer is selected to count the number of head thrashes in experimental video. The experimental results show that the counting results of the proposed algorithm are comparable to those of human observers. In previous studies, the number of head thrashes was obtained through observation and counting, which is a time-consuming and labor-intensive process. Worm head thrashes frequency is a key locomotive behaviors indicator in toxicological research. Automatic counting of head thrashes will play an important role in toxicological research.

In addition, the proposed algorithm not only counted the number of head thrashes of N2 of *C. elegans*, but also selected three long lifespan strains: *ser-1 (ok345)*, *daf-7 (m62)* and *egl-8 (n488)* of *C. elegans*, and three short lifespan strains: *daf-5 (e1386)*, *ser-4 (ok512)* and *unc-10 (md1117)* of *C. elegans*. The statistical results show that the average number of head thrashes of long-lived worms is higher than that of N2 of *C. elegans*. The average number of head thrashes of worms with short lifespan is significantly lower than that of N2 of *C. elegans*. All experimental worms have the same age, indicating that the worm with long lifespan has higher vitality. The proposed algorithm test parameters related to the number of head thrashes of worms with different lifespans, it is proved that the relationship between worm vitality and lifespan [33–39].

### Comparison to related work

To increase the recognition accuracy of worm’s head, the proposed algorithm use three criteria. The first criterion is that the head of the worm is rounder than the tail; The second criterion is that the tail of the worm is darker than the head; The third criterion is based on the head distance between two consecutive frames. FIMTrack method [40] used criterion 1 to recognize the worm’s head in each frame during the tracking process of worm movement. In the tracking process, if the head of one frame is recognized incorrectly, it will cause great errors to the final calculation result. In this paper, the proposed algorithm adds criterion 2 to criterion 1 and reduces the error rate of head recognition by 0.8%. In addition, we use criterion 3 during the tracking of worm movement. As mentioned in Ref. [22], among the four offsets of head and tail in worm coordinates for two consecutive frames, the corresponding offsets of head-head and tail–tail are the smallest. Therefore, the recognition accuracy of the worm head in each frame and algorithm efficiency could be improved.

**Fig. 7:**
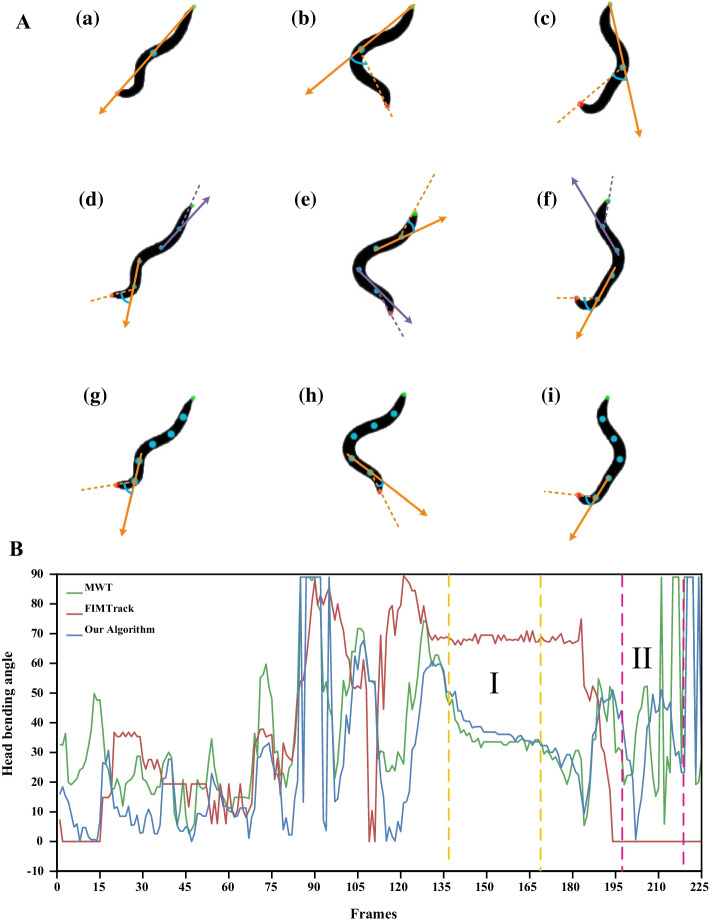
The bending angles calculated by our method and other methods. **A** The diagram for calculating the bending angles. (a–c) are FIMTrack method. (d–f) are MWT method. (g–i) are our method. The red dot represents the head; The green dots represent the tails. The orange line represents the bending angle calculated using the corresponding method. **B** Curves of bending angles. The red curve is the bending angles calculated using the FIMTrack method. The green curve is the bending angles calculated using the MWT method. The blue curve is the bending angles calculated using our method. In area I of **B**, the red curve is higher than the blue curve, indicating that the angles calculated by FIMTrack method is higher than that calculated by our method. This situation corresponds to **A** (b, c) and **A** (h, i). In area II of **B**, the red curve is a straight line with a calculated angle of 0 degrees. This situation corresponds to **A** (a). The head, midpoint of the spine and tail of the worm are in a straight line

Many systems can measure specific behavioral parameters of worms, but none can automatically count the number of head thrashes. However, some systems can calculate the bending angles. For example, the software FIMTrack in [40] proposed a method to judge the bending direction of the worm body by calculating the bending angles. FIMTrack calculated the bending angles based on the coordinates of the three points of the head, midpoint of the spine and tail. The calculated bending angles range from 0 to 360 degrees. Our method computes the bending angles by selecting three consecutive equipartition points starting from the head. The calculated bending angles range from 0 to 90 degrees. In order to compare the bending angles calculated by the two methods, we standardize the bending angles calculated by FIMTrack method. The curves of bending angles calculated by the two methods are shown in Fig. 7. As can be seen from Fig. 7A(a), when the head, midpoint and tail of worm are in a straight line, the head bending angle calculated by FIMTrack method is 180 degrees, which is 0 degrees after standardized, corresponding to area II in Fig. 7B. It can be seen from Fig. 7 that FIMTrack method cannot accurately calculate the small amplitude head thrashes behavior of worms. In addition, the system Multi-Worm Tracker (MWT) in [41] proposed a method to judge “end wiggle” of the worm by calculating the bending angles. Angle in radians between the last 20% of the body and the rest of the body (using whichever end shows a greater angle). In the calculation process, MWT method could not automatically recognize the worm’s head, but chose the end with the largest angle as the calculation result. The curves of bending angles calculated by MWT method are shown in Fig. 7. As can be seen from Fig. 7, the bending angles calculated by MWT method are higher than that calculated by our method in most cases. As can be seen from Fig. 7A(e), the bending angle of the tail is greater than that of the head, so the bending angle of the head or tail is calculated by using MWT method. Compared with MWT method, the proposed algorithm can accurately recognize the head position of worm in each frame and change the number of equipartition points according to the size of worm, thus making the calculated head bending angles more accurate. Compared with the proposed algorithm, the bending angles calculated by FIMTrack and MWT methods cannot be directly used to count head thrashes. The proposed algorithm can not only calculate the head bending angles of worms with different shapes and sizes, but also calculate the head thrashes frequency automatically.

### Applications for the proposed algorithm

The proposed algorithm is useful for automatically quantifying the head thrashes behavior of worms, which would facilitate the high-throughput forward genetic screens or drug-candidate screens using the worm [6–16]. For videos recorded under different contrast, lighting condition, background and other conditions, if worms are directly identified or unified image preprocessing methods are used, certain errors will be caused to the experimental results. Therefore, worms need to be placed on a tracking plate with a clean background and uniform light during the recording process. In addition, there can only be one worm per video. In future studies, we will consider simultaneous detection of multiple nematodes in a video to enhance the usability of the algorithm.

**Fig. 8:**
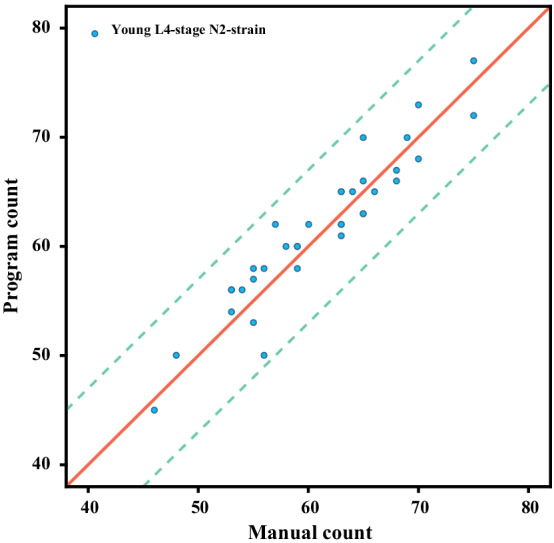
Results for counting the number of head thrashes manually and programmatically. The horizontal axis is the number of head thrashes is manually counted and vertical axis is the number of head thrashes is programmatically counted. The solid red line is a straight line with slope k = 1. The two green dotted lines are error lines with slope k = 1 and intercept b = 7

Experiments on another database [42] are conducted to prove the robustness of the proposed algorithm. More detailed information on data collection can be obtained from [43]. In brief, young L4-stage N2 of *C. elegans* were imaged with a video tracking microscope at f = 32 Hz. Worms grow at 20˚C under standard conditions [44]. First of all, the worms were removed from bacteria-laden agar plates with a platinum worm pick and rinsed with water. Let them swim in the NGM buffer for one minute. Then they were transferred to an analysis plate (a 9 cm Petri dish) containing copper rings (5.1 cm inside diameter) that were pressed into the agar surface to prevent the worms from reaching the sides of the plate. The recording starts about 5 min. After transfer, it lasts for 35 min. A total of 12 worms were recorded [42]. We randomly select three one-minute video sequences from each worm video. A total of 36 one-minute videos are selected. The proposed algorithm is used to count the number of head thrashes in each video and record the results. Experimental results of manual count and program count are shown in Fig. 8. The results of manual count and program count show the linear distribution and the average absolute error is **2.0857**, the Pearson Correlation Coefficient between manual counting results and program counting results is **0.9414**, indicating that our algorithm is very robust.

## Conclusions

In order to reduce the time and manpower consumption in toxicological studies, a new method is proposed to automatically count the number of head thrashes of worms. The robustness of the proposed algorithm is evaluated by comparing the counting results of the manual counting. It is proved that the proposed algorithm can recognize the occurrence of head thrashes of *C. elegans* of different strains. In addition, we analyze the difference of the head thrashes behavior of different worm strains, it is proved that the relationship between worm head thrashes behavior and lifespan [33–39]. The proposed algorithm will play an important role in toxicological research and worm vitality research.

## Data Availability

The code is freely available at https://github.com/hthana/HTC.
